# IMDAV reaction between phenyl­maleic anhydride and thien­yl(fur­yl)allyl­amines: synthesis and mol­ecular structure of (3a*SR*,4*RS*,4a*RS*,7a*SR*)-5-oxothieno- and (3a*SR*,4*SR*,4a*RS*,7a*SR*)-5-oxofuro[2,3-*f*]iso­indole-4-carb­oxy­lic acids

**DOI:** 10.1107/S2056989018012239

**Published:** 2018-09-07

**Authors:** Flavien A. A. Toze, Maryana A. Nadirova, Dmitriy F. Mertsalov, Julya S. Sokolova, Pavel V. Dorovatovskii, Victor N. Khrustalev

**Affiliations:** a Department of Chemistry, Faculty of Sciences, University of Douala, PO Box 24157, Douala, Republic of Cameroon; b Organic Chemistry Department, Peoples’ Friendship University of Russia (RUDN University), 6 Miklukho-Maklay St., Moscow 117198, Russian Federation; c National Research Centre "Kurchatov Institute", 1 Acad. Kurchatov Sq., Moscow 123182, Russian Federation; d Inorganic Chemistry Department, Peoples’ Friendship University of Russia (RUDN University), 6 Miklukho-Maklay St., Moscow 117198, Russian Federation

**Keywords:** IMDAV reaction, acid anhydrides, thien­yl(fur­yl)allyl­amines, iso­indole-4-carb­oxy­lic acids, crystal structure, synchrotron radiation, hydrogen bonds, disorder

## Abstract

The title compounds C_24_H_21_NO_3_S, **I**, and C_24_H_21_NO_4_, **II**, are the products of the IMDAV reaction between phenyl­maleic anhydride and thien­yl(fur­yl)allyl­amines. Their mol­ecular structures comprise fused tricyclic systems containing thio­phene, cyclo­hexene and pyrrolidine rings (**I**) or furan, cyclo­hexene and pyrrolidine rings (**II**). The central cyclo­hexene and pyrrolidine rings in both compounds adopt slightly twisted boat and envelope conformations, respectively. The dihedral angles between the basal plane of the pyrrolidine ring and the thio­phene (in **I**) or furan (in **II**) ring plane are 22.74 (16) and 26.29 (5)°, respectively. The nitro­gen atom both in **I** and **II** has practically planar environment [the sums of the bond angles are 359.8 and 358.9°, respectively]. In the crystal of **I**, the mol­ecules form hydrogen-bonded zigzag chains along [010] through strong inter­molecular O—H⋯O hydrogen bonds involving carb­oxy­lic and keto groups, whereas in the crystal of **II**, the mol­ecules are joined into centrosymmetric dimers by strong O—H⋯O hydrogen bonds between the carb­oxy­lic groups. In **II**, the atoms involved into these hydrogen bonds (and hence the whole carb­oxy­lic group) are disordered over two sets of sites with an occupancy ratio of 0.6:0.4. Compounds **I** and **II** crystallize as racemates consisting of enanti­omeric pairs of the 3a*SR*,4*RS*,4a*RS*,7a*SR* and 3a*SR*,4*SR*,4a*RS*,7a*SR* diastereomers, respectively.

## Abstract 2

To establish the scope and limitations of the IMDAV reaction by elucidation of its regio- and stereoselectivity, the products of the reaction between phenyl­maleic anhydride and thien­yl(fur­yl)allyl­amines were studied by X-ray diffraction

## Chemical context   

Cascade transformations including one or more tandem or sequential [4 + 2] cyclo­addition reactions are a useful and high-usage tool in organic synthesis (Parvatkar *et al.*, 2014 ▸; Sears & Boger, 2016 ▸; Borisova *et al.*, 2018 ▸). In most cases, conjugated linear or cyclic alkadienes are the starting mat­erials for these transformations. Along with this, it has long been known that furan, thio­phene and pyrrole, possessing a conjugated system of double bonds, can also act as a diene moiety. Around 50 years ago, it was found that 2-vinyl­furans and 2-vinyl­thio­phenes can play the role of dienes in the inter­molecular Diels–Alder reaction, which cleared a short way to benzo­furan or benzo­thio­phene derivatives (Paul, 1943 ▸; Szmuszkovicz & Modest, 1950 ▸; Schmidt, 1953 ▸; Scully & Brown, 1953 ▸; Davies & Porter, 1957*a* ▸,*b* ▸; Kaufmann & Sen Gupta, 1963 ▸; Ancerewicz & Vogel, 1993 ▸; Drew *et al.*, 2002 ▸; Wavrin *et al.*, 2004 ▸; Ghobsi *et al.*, 2008 ▸). At the end of the last century, it was demonstrated that this reaction could be performed in an intra­molecular variant when both a heterocyclic diene and a dienophilic moiety are incorporated in the same mol­ecule.

**Figure 1:**
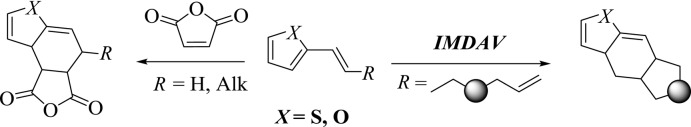
Intra- and inter­molecular Diels–Alder reaction in vinyl­furans and vinyl­thio­phenes in the synthesis of benzo­furans and benzo­thio­phenes.

The IMDAV (**I**ntra**M**olecular **D**iels–**A**lder **V**inylarenes) reaction (Fig. 1 ▸) has become a powerful tool in organic synthesis because of its simplicity and reliability, which assures good yields of benzo­furans and benzo­thio­phenes annulated with other carbo- and heterocycles (Maas *et al.*, 2006 ▸; Patre *et al.*, 2007 ▸; Kim *et al.*, 2014 ▸).

**Figure 2:**
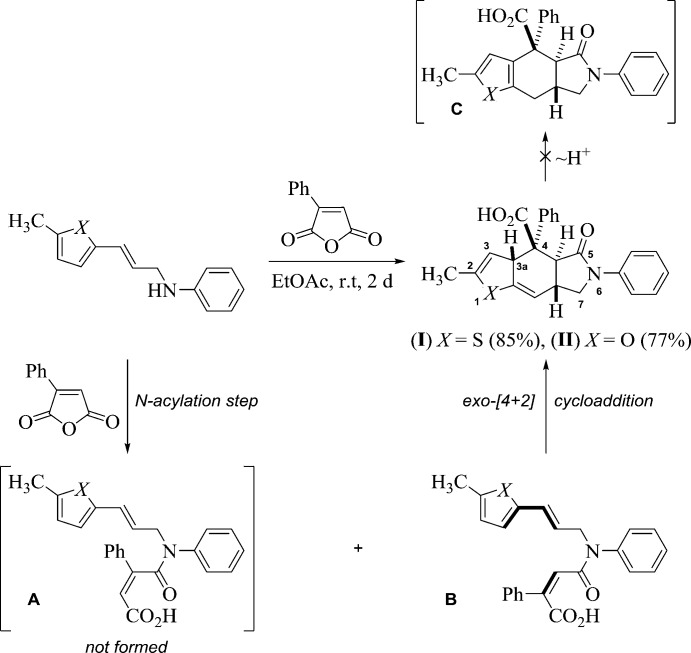
Synthesis of (3a*SR*,4*RS*,4a*RS*,7a*SR*)-5-oxothieno[2,3-*f*]iso­indole-4-carb­oxy­lic acid (I) and (3a*SR*,4*SR*,4a*RS*,7a*SR*)-5-oxofuro[2,3-*f*]iso­indole-4-carb­oxy­lic acid (II).

Previously, with the example of the inter­action between maleic anhydride and 3-thien­yl(fur­yl)allyl­amines, our group demonstrated the possibility of the domino-sequence involving *N*-acyl­ation, IMDAV reaction and aromatization steps leading to 4*H*-furo- or thieno[2,3-*f*]iso­indoles (Horak *et al.*, 2015 ▸, 2017 ▸; Zubkov *et al.*, 2016 ▸). The aim of the present study was elucidation of the regio- and stereoselectivity of the reaction between phenyl­maleic anhydride and thien­yl(fur­yl)allyl­amines in order to establish the scope and the limitations of the IMDAV reaction (Fig. 2 ▸).

The reaction proceeds smoothly at room temperature, a simple filtration of the resulting crystalline products from ethyl acetate giving adducts **I** and **II** in good yields. The Diels–Alder reaction proceeds regio- and stereoselectively as an *exo*-[4 + 2] cyclo­addition (Fig. 2 ▸). The nucleophilic attack of the nitro­gen atom is directed at the least sterically hindered carbon atom of the carbonyl group of phenyl­maleic anhydride, thus amide **A** is not formed. The inter­mediate amide **B** cannot be isolated, and the spontaneous intra­molecular Diels–Alder reaction completes the process, leading to the target compounds **I** and **II**. The migration of proton H3a in adducts **I**, **II** and the formation of compound **C** is not observed under these conditions (Horak *et al.*, 2015 ▸, 2017 ▸; Zubkov *et al.*, 2016 ▸).
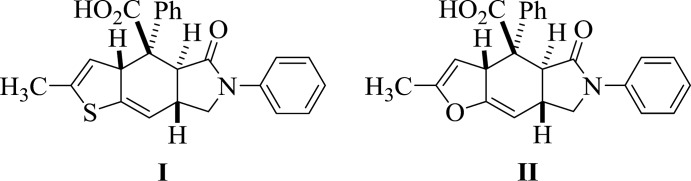


## Structural commentary   

Despite the very similar mol­ecular structures, compounds **I**, C_24_H_21_NO_3_S and **II**, C_24_H_21_NO_4_ are not isostructural. Compound **I** crystallizes in the monoclinic space group *P*2_1_/n, while compound **II** crystallizes in the triclinic space group *P* 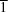.

**Figure 3:**
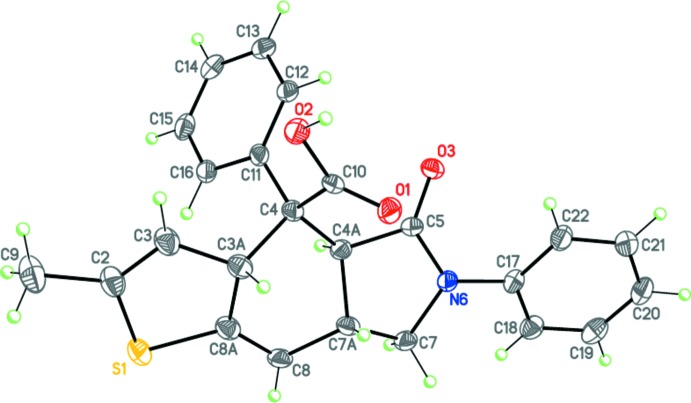
Mol­ecular structure of **I**. Displacement ellipsoids are shown at the 50% probability level. H atoms are presented as small spheres of arbitrary radius.

**Figure 4:**
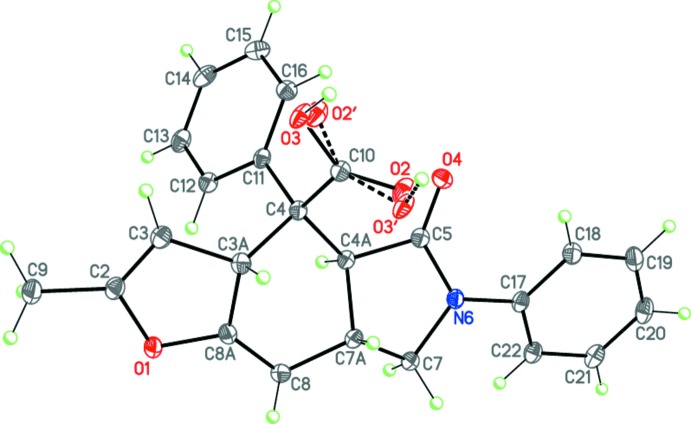
Mol­ecular structure of **II**. Displacement ellipsoids are shown at the 50% probability level. H atoms are presented as small spheres of arbitrary radius. The minor occupancy position of the –COOH group is depicted with dashed lines.

The mol­ecules of **I** and **II** comprise fused tricyclic systems containing thio­phene, cyclo­hexene and pyrrolidine rings in **I** (Fig. 3 ▸) and furan, cyclo­hexene and pyrrolidine rings in **II** (Fig. 4 ▸). The central cyclo­hexene and pyrrolidine rings in both compounds adopt slightly distorted boat and envelope conformations, respectively. The dihedral angles between the basal plane of the pyrrolidine ring (N5/N6/C4*A*/C7) and the thio­phene (in **I**) or furan (in **II**) ring planes are 22.74 (16) and 26.29 (5)°, respectively. The N6 nitro­gen atom both in **I** and **II** has practically planar environment (the sums of the bond angles are 359.8 and 358.9°, respectively).

In the mol­ecule of **II**, the carb­oxy­lic group is disordered over two orientations with inter­changing hydrogen atom positions (Fig. 4 ▸), the occupancy ratio being 0.6:0.4.

The mol­ecules of **I** and **II** possess four asymmetric centers at the C3*A*, C4, C4*A* and C7*A* carbon atoms and potentially can have numerous diastereomers. The crystals of **I** and **II** are racemic and consist of enanti­omeric pairs with the following relative configuration of the centers: 3a*SR*,4*RS*,4a*RS*,7a*SR* and 3a*SR*,4*SR*,4a*RS*,7a*SR*, respectively, thus **I** and **II** differ in the configuration at the C4 atom.

## Supra­molecular features   

**Figure 5:**
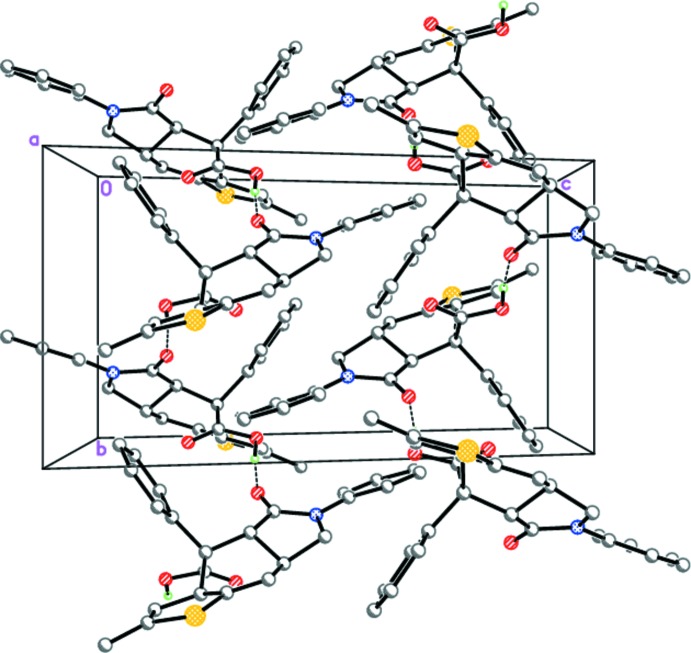
The hydrogen-bonded zigzag chains along the *b-*axis direction in **I**. Dashed lines indicate the inter­molecular O—H⋯O hydrogen bonds.

In the crystal of **I**, mol­ecules form hydrogen-bonded zigzag chains propagating along [010] through strong O—H⋯O hydrogen bonds involving the carb­oxy­lic and keto groups (Table 1 ▸, Fig. 5 ▸).

**Figure 6:**
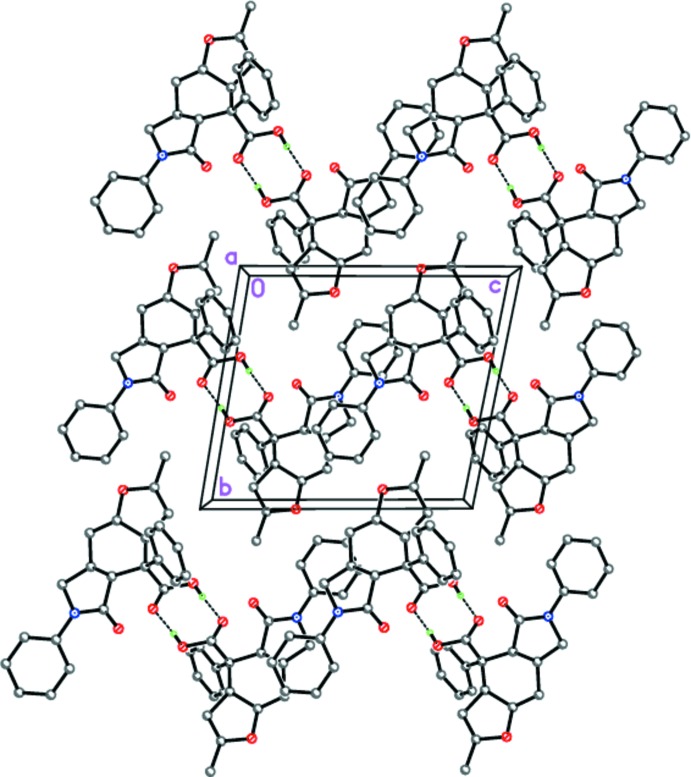
The hydrogen-bonded centrosymmetric dimers of **II**. Dashed lines indicate the inter­molecular O—H⋯O hydrogen bonds. The minor occupancy –COOH groups are omitted for clarity.

Contrary to **I**, in the crystal of **II**, mol­ecules form hydrogen-bonded centrosymmetric dimers through pairs of strong O—H⋯O hydrogen bonds between two carb­oxy­lic groups (Table 2 ▸, Fig. 6 ▸). The dimers are stacked along the *a**-***axis direction.

## Synthesis and crystallization   

2-Methyl-4,6-diphenyl-4,4a,5,6,7,7a-hexa­hydro-3a*H*-thieno(furo)[2,3-*f*]iso­indole-4-carb­oxy­lic acids (**I** and **II**) were synthesized using a method similar to the procedure described recently (Horak *et al.*, 2015 ▸, 2017 ▸; Zubkov *et al.*, 2016 ▸).

**General procedure.** A solution of *N*-[(2*E*)-3-(5-methyl­thio­phen-2-yl)prop-2-en-1-yl]aniline (for **I**) or *N*-[(2*E*)-3-(5-methyl­furan-2-yl)prop-2-en-1-yl]aniline (for **II**) (2 mmol) in ethyl acetate (10 mL) was placed into a 25 mL round-bottom flask and then phenyl­maleic anhydride (0.35 g, 2.0 mmol) was added. The mixture was stirred for two days at room temperature. The formed precipitate was filtered off, washed with Et_2_O (2 × 10 mL) and dried in air. The resulting product was recrystallized from a mixture of EtOH–DMF (5:1 *v*:*v*) to afford the analytically pure samples of target products.

**(3a** ***RS*** **,4** ***SR*** **,4a** ***SR*** **,7a** ***SR*** **)-2-Methyl-5-oxo-4,6-diphenyl-4,4a,5,6,7,7a-hexa­hydro-3a** ***H*** **-thieno[2,3-** ***f*** **]iso­indole-4-carb­oxy­lic acid (I).** Colourless prisms. Yield 0.69 g (85%). M.p. = 447.1–448.1 K. IR (KBr), ν (cm^−1^): 3095, 1701. ^1^H NMR (DMSO-*d*_6_, 600.2 MHz, 301 K) *δ* = 13.04 (*s*, 1H, CO_2_H), 7.52–7.03 (*m*, 10H, HAr), 6.30 (*dt*, 1H, H8, *J* = 1.0, *J* = 3.5), 5.15 (*pent*, 1H, H3, *J* = 1.3), 4.16–4.14 (*m*, 1H, H3a), 3.99 (*dd*, 1H, H7a*, J* = 7.6, *J* = 8.8), 3.67 (*dd*, 1H, H7b*, J* = 8.8, *J* = 10.8), 2.95–2.89 (*m*, 1H, H7a), 2.25 (*d*, 1H, H4a, *J* = 12.6), 1.92 (*q*, 3H, CH_3_, *J* = 1.3). ^13^C NMR (DMSO-*d*_6_, 150.9 MHz, 301 K): δ = 175.4, 171.2 (CO_2_, NCO), 143.1, 141.3, 140.4, 136.6, 129.1 (2C), 129.0 (2C), 127.7 (2C), 126.5, 124.1, 120.5, 120.3, 119.7 (2C), 61.9, 60.1, 54.7, 49.5, 37.9, 16.7 (CH_3_). MS (APCI): *m*/*z* = 404 [*M* + H]^+^.

**(3a** ***RS*** **,4** ***RS*** **,4a** ***SR*** **,7a** ***RS*** **)-2-Methyl-5-oxo-4,6-diphenyl-4,4a,5,6,7,7a-hexa­hydro-3a** ***H*** **-furo[2,3-** ***f*** **]iso­indole-4-carb­oxy­lic acid (II).** Colourless prisms. Yield 0.60 g (77%). M.p. = 422-423 K. IR (KBr), ν (cm^−1^): 1703, 1656. ^1^H NMR (DMSO-*d*_6_, 600.2 MHz, 301 K) *δ* = 13.00 (*s*, 1H, CO_2_H), 7.55 (*dd*, 2H, HAr, *J* = 7.6, *J* = 8.3), 7.33 (*dd*, 2H, HAr, *J* = 7.6, *J* = 8.6), 7.24 (*dd*, 2H, HAr, *J* = 7.6, *J* = 8.3), 7.15–7.13 (*m*, 3H, HAr), 7.08 (*t*, 1H, HAr, *J* = 7.6), 5.59 (*dt*, 1H, H8, *J* = 1.0, *J* = 3.5), 4.68 (*dd*, 1H, H3a, *J* = 1.0, *J* = 1.5), 4.08–4.02 (*m*, 2H, H3, H7a), 3.68 (*dd*, 1H, H7b*, J* = 8.8, *J* = 10.8), 2.94–2.88 (*m*, 1H, H7a), 2.40 (*d*, 1H, H4a, *J* = 12.1), 1.91 (*s*, 3H, CH_3_). ^13^C NMR (DMSO-*d*_6_, 150.9 MHz, 301 K): δ = 174.8, 170.7 (CO_2_, NCO), 157.8, 154.3, 141.9, 139.9, 128.8 (2C), 128.5 (2C), 127.1 (2C), 125.9, 123.5, 119.1 (2C), 100.2, 97.6, 60.9, 53.4, 52.5, 49.3, 35.1, 13.3 (CH_3_). MS (APCI): *m*/*z* = 388 [*M* + H]^+^.

## Refinement   

Crystal data, data collection and structure refinement details are summarized in Table 3 ▸. X-ray diffraction studies were carried out on the ‘Belok’ beamline of the National Research Center ‘Kurchatov Institute’ (Moscow, Russian Federation) using a Rayonix SX165 CCD detector. A total of 720 images for each compounds were collected using an oscillation range of 1.0° (*φ* scan mode, two different crystal orientations) and corrected for absorption using the *SCALA* program (Evans, 2006 ▸). The data were indexed, integrated and scaled using the utility *iMOSFLM* in the CCP4 program (Battye *et al.*, 2011 ▸).

The COOH-group in **II** is disordered over two orientations. The refinement of their occupancy factors was unstable, thus the occupancies were constrained to a 0.6:0.4 ratio. The two positions of this group were refined at fixed C=O and C—O distances of 1.210 (3) and 1.320 (3) Å, respectively. Moreover, the anisotropic displacement parameters for the oxygen atoms of the C=O and C—O groups were restrained to be equal.

The hydrogen atoms of the OH groups were localized in difference-Fourier maps and refined isotropically with fixed displacement parameters [*U*_iso_(H) = 1.5*U*_eq_(O)]. The other hydrogen atoms were placed in calculated positions with C—H = 0.95–1.00 Å and refined using the riding model with fixed isotropic displacement parameters [*U*_iso_(H) = 1.5*U*_eq_(C) for the CH_3_ groups and 1.2*U*_eq_(C) for all others].

A relatively large number of reflections (a few dozen) were omitted for the following reasons: (1) In order to achieve better *I*/σ statistics for high-angle reflections we selected a larger exposure time, which resulted in some intensity overloads in the low-angle part of the area. These corrupted intensities were excluded from the final steps of the refinement. (2) In the current setup of the instrument, the low-temperature device eclipses a small region of the detector near its high-angle limit. This resulted in zero intensity for some reflections. (3) The quality of the single crystals chosen for the diffraction experiments was far from perfect. Some systematic intensity deviations can be due to extinction and defects present in the crystals.

## Supplementary Material

Crystal structure: contains datablock(s) global, I, II. DOI: 10.1107/S2056989018012239/yk2117sup1.cif

Structure factors: contains datablock(s) I. DOI: 10.1107/S2056989018012239/yk2117Isup2.hkl

Structure factors: contains datablock(s) II. DOI: 10.1107/S2056989018012239/yk2117IIsup3.hkl

CCDC references: 1864385, 1864384

Additional supporting information:  crystallographic information; 3D view; checkCIF report

## Figures and Tables

**Table 1:** Hydrogen-bond geometry (Å, °) for **I** [Chem scheme1]

*D*—H⋯*A*	*D*—H	H⋯*A*	*D*⋯*A*	*D*—H⋯*A*
O2—H2⋯O3^i^	1.04 (5)	1.63 (5)	2.667 (4)	174 (4)

Symmetry code: (i) 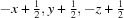.

**Table 2:** Hydrogen-bond geometry (Å, °) for **II** [Chem scheme1]

*D*—H⋯*A*	*D*—H	H⋯*A*	*D*⋯*A*	*D*—H⋯*A*
O3—H3*B*⋯O2^i^	0.91 (3)	1.79 (3)	2.692 (3)	176 (3)
O3′—H3*C*⋯O2′^i^	0.91 (5)	1.79 (5)	2.690 (6)	169 (4)

Symmetry code: (i) 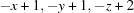.

**Table 3:** Experimental details

	**I**	**II**
Crystal data		
Chemical formula	C_24_H_21_NO_3_S	C_24_H_21_NO_4_
*M*_r_	403.48	387.42
Crystal system, space group	Monoclinic, *P*2_1_/*n*	Triclinic, *P* 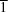
Temperature (K)	100	100
*a*, *b*, *c* (Å)	14.572 (3), 8.7989 (18), 16.982 (3)	8.1851 (16), 11.025 (2), 11.795 (2)
α, β, γ (°)	90, 111.92 (3), 90	99.14 (3), 92.51 (3), 107.99 (3)
*V* (Å^3^)	2020.0 (8)	994.6 (4)
*Z*	4	2
Radiation type	Synchrotron, λ = 0.96260 Å	Synchrotron, λ = 0.81182 Å
μ (mm^−1^)	0.41	0.12
Crystal size (mm)	0.15 × 0.10 × 0.10	0.20 × 0.12 × 0.08

Data collection		
Diffractometer	Rayonix SX165 CCD	Rayonix SX165 CCD
Absorption correction	Multi-scan (*SCALA*; Evans, 2006 ▸)	Multi-scan (*SCALA*; Evans, 2006 ▸)
*T*_min_, *T*_max_	0.930, 0.950	0.963, 0.987
No. of measured, independent and observed [*I* > 2σ(*I*)] reflections	12006, 4140, 2888	18107, 4204, 3839
*R*_int_	0.101	0.092
(sin θ/λ)_max_ (Å^−1^)	0.645	0.634

Refinement		
*R*[*F* ^2^ > 2σ(*F* ^2^)], *wR*(*F* ^2^), *S*	0.082, 0.244, 1.04	0.048, 0.129, 1.06
No. of reflections	4140	4204
No. of parameters	267	276
No. of restraints	0	4
H-atom treatment	H atoms treated by a mixture of independent and constrained refinement	H atoms treated by a mixture of independent and constrained refinement
Δρ_max_, Δρ_min_ (e Å^−3^)	0.57, −0.83	0.31, −0.25

Computer programs: *MarCCD* (Doyle, 2011 ▸), *iMosflm* (Battye *et al.*, 2011 ▸), *SHELXT* (Sheldrick, 2015*a* ▸), *SHELXL2014* (Sheldrick, 2015*b* ▸) and *SHELXTL* (Sheldrick, 2008 ▸).

## supplementary crystallographic information

### (3aSR,4RS,4aRS,7aSR)-5-Oxothieno[2,3-f]isoindole-4-carboxylic acid (I) Crystal data

**Table:**

C_24_H_21_NO_3_S	*F*(000) = 848
*M**_r_* = 403.48	*D*_x_ = 1.327 Mg m^−^^3^
Monoclinic, *P*2_1_/*n*	Synchrotron radiation, λ = 0.96260 Å
*a* = 14.572 (3) Å	Cell parameters from 600 reflections
*b* = 8.7989 (18) Å	θ = 3.6–36.0°
*c* = 16.982 (3) Å	µ = 0.41 mm^−^^1^
β = 111.92 (3)°	*T* = 100 K
*V* = 2020.0 (8) Å^3^	Prism, colourless
*Z* = 4	0.15 × 0.10 × 0.10 mm

### (3aSR,4RS,4aRS,7aSR)-5-Oxothieno[2,3-f]isoindole-4-carboxylic acid (I) Data collection

**Table:**

Rayonix SX165 CCD diffractometer	2888 reflections with *I* > 2σ(*I*)
/f scan	*R*_int_ = 0.101
Absorption correction: multi-scan (SCALA; Evans, 2006)	θ_max_ = 38.4°, θ_min_ = 3.6°
*T*_min_ = 0.930, *T*_max_ = 0.950	*h* = −12→18
12006 measured reflections	*k* = −10→8
4140 independent reflections	*l* = −21→14

### (3aSR,4RS,4aRS,7aSR)-5-Oxothieno[2,3-f]isoindole-4-carboxylic acid (I) Refinement

**Table:**

Refinement on *F*^2^	Secondary atom site location: difference Fourier map
Least-squares matrix: full	Hydrogen site location: mixed
*R*[*F*^2^ > 2σ(*F*^2^)] = 0.082	H atoms treated by a mixture of independent and constrained refinement
*wR*(*F*^2^) = 0.244	*w* = 1/[σ^2^(*F*_o_^2^) + (0.08*P*)^2^ + 4*P*] where *P* = (*F*_o_^2^ + 2*F*_c_^2^)/3
*S* = 1.04	(Δ/σ)_max_ < 0.001
4140 reflections	Δρ_max_ = 0.57 e Å^−^^3^
267 parameters	Δρ_min_ = −0.83 e Å^−^^3^
0 restraints	Extinction correction: SHELXL (Sheldrick, 2015b), Fc^*^=kFc[1+0.001xFc^2^λ^3^/sin(2θ)]^-1/4^
Primary atom site location: difference Fourier map	Extinction coefficient: 0.027 (4)

### (3aSR,4RS,4aRS,7aSR)-5-Oxothieno[2,3-f]isoindole-4-carboxylic acid (I) Special details

**Table:**

Geometry. All esds (except the esd in the dihedral angle between two l.s. planes) are estimated using the full covariance matrix. The cell esds are taken into account individually in the estimation of esds in distances, angles and torsion angles; correlations between esds in cell parameters are only used when they are defined by crystal symmetry. An approximate (isotropic) treatment of cell esds is used for estimating esds involving l.s. planes.

### (3aSR,4RS,4aRS,7aSR)-5-Oxothieno[2,3-f]isoindole-4-carboxylic acid (I) Fractional atomic coordinates and isotropic or equivalent isotropic displacement parameters (Å^2^)

**Table:**

	*x*	*y*	*z*	*U*_iso_*/*U*_eq_	
S1	0.70674 (7)	0.54365 (14)	0.24110 (6)	0.0381 (4)	
C2	0.6019 (3)	0.5821 (5)	0.1467 (3)	0.0360 (10)	
C3	0.5152 (3)	0.5575 (5)	0.1543 (2)	0.0337 (9)	
H3	0.4540	0.5694	0.1079	0.040*	
C3A	0.5203 (3)	0.5094 (5)	0.2417 (2)	0.0288 (8)	
H3A	0.4993	0.6007	0.2658	0.035*	
C4	0.4494 (2)	0.3754 (4)	0.2477 (2)	0.0253 (8)	
C4A	0.4981 (2)	0.3035 (4)	0.3372 (2)	0.0249 (8)	
H4A	0.5376	0.2153	0.3299	0.030*	
C5	0.4338 (3)	0.2403 (4)	0.3820 (2)	0.0243 (8)	
O3	0.35066 (18)	0.1829 (3)	0.34752 (14)	0.0299 (7)	
N6	0.4879 (2)	0.2501 (4)	0.46806 (17)	0.0274 (7)	
C7	0.5884 (3)	0.3135 (5)	0.4862 (2)	0.0295 (9)	
H7A	0.6378	0.2318	0.4942	0.035*	
H7B	0.6097	0.3795	0.5371	0.035*	
C7A	0.5723 (2)	0.4056 (4)	0.4050 (2)	0.0248 (8)	
H7C	0.5384	0.5029	0.4081	0.030*	
C8	0.6579 (3)	0.4419 (5)	0.3776 (2)	0.0305 (9)	
H8	0.7252	0.4334	0.4146	0.037*	
C8A	0.6300 (3)	0.4869 (5)	0.2967 (2)	0.0302 (9)	
C9	0.6196 (4)	0.6459 (6)	0.0702 (3)	0.0494 (12)	
H9A	0.6507	0.7462	0.0844	0.074*	
H9B	0.5563	0.6553	0.0223	0.074*	
H9C	0.6633	0.5773	0.0549	0.074*	
C10	0.3550 (3)	0.4624 (4)	0.2411 (2)	0.0243 (8)	
O1	0.33975 (18)	0.5090 (3)	0.30274 (15)	0.0303 (7)	
O2	0.29599 (19)	0.4951 (3)	0.16006 (15)	0.0294 (6)	
H2	0.240 (3)	0.567 (5)	0.161 (3)	0.044*	
C11	0.4327 (2)	0.2480 (5)	0.1814 (2)	0.0258 (8)	
C12	0.3445 (3)	0.1640 (4)	0.1514 (2)	0.0263 (8)	
H12	0.2902	0.1956	0.1656	0.032*	
C13	0.3348 (3)	0.0346 (5)	0.1011 (2)	0.0305 (9)	
H13	0.2747	−0.0213	0.0819	0.037*	
C14	0.4136 (3)	−0.0122 (5)	0.0790 (2)	0.0326 (9)	
H14	0.4070	−0.0992	0.0441	0.039*	
C15	0.5016 (3)	0.0688 (5)	0.1082 (2)	0.0338 (10)	
H15	0.5554	0.0364	0.0936	0.041*	
C16	0.5118 (3)	0.1985 (4)	0.1591 (2)	0.0270 (8)	
H16	0.5724	0.2531	0.1786	0.032*	
C17	0.4580 (3)	0.1950 (4)	0.5344 (2)	0.0270 (8)	
C18	0.5317 (3)	0.1501 (5)	0.6121 (2)	0.0324 (9)	
H18	0.5993	0.1491	0.6185	0.039*	
C19	0.5044 (3)	0.1070 (5)	0.6798 (2)	0.0383 (10)	
H19	0.5539	0.0761	0.7320	0.046*	
C20	0.4059 (3)	0.1088 (5)	0.6717 (2)	0.0372 (10)	
H20	0.3882	0.0816	0.7183	0.045*	
C21	0.3333 (3)	0.1510 (5)	0.5943 (2)	0.0362 (10)	
H21	0.2658	0.1505	0.5882	0.043*	
C22	0.3584 (3)	0.1941 (5)	0.5253 (2)	0.0323 (9)	
H22	0.3083	0.2225	0.4729	0.039*	

### (3aSR,4RS,4aRS,7aSR)-5-Oxothieno[2,3-f]isoindole-4-carboxylic acid (I) Atomic displacement parameters (Å^2^)

**Table:**

	*U*^11^	*U*^22^	*U*^33^	*U*^12^	*U*^13^	*U*^23^
S1	0.0248 (6)	0.0533 (8)	0.0398 (6)	−0.0065 (5)	0.0164 (5)	0.0001 (5)
C2	0.030 (2)	0.041 (2)	0.042 (2)	0.0030 (19)	0.0195 (18)	0.0056 (18)
C3	0.024 (2)	0.041 (3)	0.037 (2)	0.0027 (18)	0.0123 (17)	0.0063 (17)
C3A	0.0218 (18)	0.032 (2)	0.0324 (18)	0.0015 (16)	0.0094 (15)	0.0003 (16)
C4	0.0166 (17)	0.035 (2)	0.0225 (15)	0.0000 (15)	0.0049 (14)	0.0036 (15)
C4A	0.0176 (17)	0.034 (2)	0.0223 (16)	0.0016 (16)	0.0063 (14)	−0.0051 (15)
C5	0.0204 (17)	0.028 (2)	0.0212 (15)	−0.0020 (15)	0.0043 (14)	−0.0019 (14)
O3	0.0221 (14)	0.0400 (17)	0.0245 (12)	−0.0068 (12)	0.0053 (10)	0.0024 (11)
N6	0.0190 (15)	0.043 (2)	0.0190 (13)	−0.0002 (14)	0.0056 (12)	−0.0012 (13)
C7	0.0196 (18)	0.042 (2)	0.0233 (16)	−0.0007 (17)	0.0041 (14)	−0.0066 (16)
C7A	0.0179 (17)	0.027 (2)	0.0270 (17)	−0.0019 (15)	0.0056 (14)	−0.0040 (15)
C8	0.0168 (17)	0.042 (2)	0.0301 (18)	−0.0045 (16)	0.0053 (15)	−0.0097 (16)
C8A	0.0248 (19)	0.031 (2)	0.0369 (19)	−0.0017 (17)	0.0136 (16)	−0.0052 (16)
C9	0.047 (3)	0.053 (3)	0.056 (3)	0.000 (2)	0.029 (2)	0.017 (2)
C10	0.0163 (17)	0.032 (2)	0.0226 (15)	−0.0054 (15)	0.0052 (14)	−0.0008 (14)
O1	0.0252 (14)	0.0393 (17)	0.0279 (12)	0.0035 (12)	0.0117 (11)	−0.0030 (11)
O2	0.0220 (13)	0.0398 (17)	0.0251 (12)	0.0064 (12)	0.0073 (10)	0.0021 (11)
C11	0.0198 (17)	0.038 (2)	0.0194 (15)	0.0005 (16)	0.0070 (13)	0.0056 (15)
C12	0.0221 (18)	0.034 (2)	0.0213 (15)	0.0016 (16)	0.0066 (14)	0.0011 (14)
C13	0.029 (2)	0.036 (2)	0.0223 (16)	0.0002 (17)	0.0043 (15)	0.0001 (15)
C14	0.042 (2)	0.034 (2)	0.0204 (16)	0.0033 (19)	0.0101 (16)	−0.0011 (15)
C15	0.035 (2)	0.045 (3)	0.0249 (17)	0.0068 (19)	0.0162 (17)	0.0013 (17)
C16	0.0240 (19)	0.035 (2)	0.0233 (16)	0.0004 (16)	0.0099 (14)	0.0027 (15)
C17	0.031 (2)	0.029 (2)	0.0221 (16)	0.0044 (16)	0.0110 (15)	−0.0002 (14)
C18	0.032 (2)	0.037 (2)	0.0258 (17)	0.0047 (18)	0.0074 (16)	−0.0002 (16)
C19	0.050 (3)	0.036 (2)	0.0252 (18)	0.005 (2)	0.0096 (18)	0.0008 (16)
C20	0.052 (3)	0.038 (2)	0.0273 (18)	0.000 (2)	0.0204 (18)	−0.0008 (16)
C21	0.037 (2)	0.040 (3)	0.035 (2)	−0.0058 (19)	0.0174 (18)	−0.0024 (17)
C22	0.035 (2)	0.038 (2)	0.0254 (17)	0.0021 (18)	0.0133 (16)	−0.0007 (16)

### (3aSR,4RS,4aRS,7aSR)-5-Oxothieno[2,3-f]isoindole-4-carboxylic acid (I) Geometric parameters (Å, º)

**Table:**

S1—C8A	1.782 (4)	C9—H9B	0.9800
S1—C2	1.785 (4)	C9—H9C	0.9800
C2—C3	1.335 (5)	C10—O1	1.219 (4)
C2—C9	1.523 (6)	C10—O2	1.353 (4)
C3—C3A	1.519 (5)	O2—H2	1.04 (5)
C3—H3	0.9500	C11—C12	1.404 (5)
C3A—C8A	1.534 (5)	C11—C16	1.409 (5)
C3A—C4	1.596 (5)	C12—C13	1.399 (5)
C3A—H3A	1.0000	C12—H12	0.9500
C4—C10	1.542 (5)	C13—C14	1.397 (5)
C4—C11	1.543 (5)	C13—H13	0.9500
C4—C4A	1.551 (5)	C14—C15	1.387 (6)
C4A—C5	1.517 (5)	C14—H14	0.9500
C4A—C7A	1.541 (5)	C15—C16	1.406 (5)
C4A—H4A	1.0000	C15—H15	0.9500
C5—O3	1.239 (4)	C16—H16	0.9500
C5—N6	1.379 (4)	C17—C22	1.400 (5)
N6—C17	1.435 (4)	C17—C18	1.412 (5)
N6—C7	1.488 (4)	C18—C19	1.401 (5)
C7—C7A	1.540 (5)	C18—H18	0.9500
C7—H7A	0.9900	C19—C20	1.390 (6)
C7—H7B	0.9900	C19—H19	0.9500
C7A—C8	1.519 (5)	C20—C21	1.394 (6)
C7A—H7C	1.0000	C20—H20	0.9500
C8—C8A	1.340 (5)	C21—C22	1.404 (5)
C8—H8	0.9500	C21—H21	0.9500
C9—H9A	0.9800	C22—H22	0.9500
			
C8A—S1—C2	91.87 (18)	C3A—C8A—S1	111.1 (3)
C3—C2—C9	127.6 (4)	C2—C9—H9A	109.5
C3—C2—S1	114.0 (3)	C2—C9—H9B	109.5
C9—C2—S1	118.3 (3)	H9A—C9—H9B	109.5
C2—C3—C3A	115.9 (4)	C2—C9—H9C	109.5
C2—C3—H3	122.1	H9A—C9—H9C	109.5
C3A—C3—H3	122.1	H9B—C9—H9C	109.5
C3—C3A—C8A	106.9 (3)	O1—C10—O2	123.5 (3)
C3—C3A—C4	118.1 (3)	O1—C10—C4	123.2 (3)
C8A—C3A—C4	114.8 (3)	O2—C10—C4	113.0 (3)
C3—C3A—H3A	105.3	C10—O2—H2	108 (2)
C8A—C3A—H3A	105.3	C12—C11—C16	118.0 (3)
C4—C3A—H3A	105.3	C12—C11—C4	121.4 (3)
C10—C4—C11	114.5 (3)	C16—C11—C4	120.0 (3)
C10—C4—C4A	110.1 (3)	C13—C12—C11	121.3 (3)
C11—C4—C4A	107.9 (3)	C13—C12—H12	119.3
C10—C4—C3A	102.1 (3)	C11—C12—H12	119.3
C11—C4—C3A	114.8 (3)	C14—C13—C12	119.9 (4)
C4A—C4—C3A	107.0 (3)	C14—C13—H13	120.0
C5—C4A—C7A	103.3 (3)	C12—C13—H13	120.0
C5—C4A—C4	119.9 (3)	C15—C14—C13	119.7 (4)
C7A—C4A—C4	115.5 (3)	C15—C14—H14	120.2
C5—C4A—H4A	105.6	C13—C14—H14	120.2
C7A—C4A—H4A	105.6	C14—C15—C16	120.5 (3)
C4—C4A—H4A	105.6	C14—C15—H15	119.7
O3—C5—N6	126.7 (3)	C16—C15—H15	119.7
O3—C5—C4A	126.1 (3)	C15—C16—C11	120.5 (3)
N6—C5—C4A	107.1 (3)	C15—C16—H16	119.8
C5—N6—C17	126.1 (3)	C11—C16—H16	119.8
C5—N6—C7	111.8 (3)	C22—C17—C18	119.7 (3)
C17—N6—C7	121.9 (3)	C22—C17—N6	121.4 (3)
N6—C7—C7A	101.7 (3)	C18—C17—N6	118.7 (3)
N6—C7—H7A	111.4	C19—C18—C17	119.6 (4)
C7A—C7—H7A	111.4	C19—C18—H18	120.2
N6—C7—H7B	111.4	C17—C18—H18	120.2
C7A—C7—H7B	111.4	C20—C19—C18	120.9 (4)
H7A—C7—H7B	109.3	C20—C19—H19	119.5
C8—C7A—C7	121.1 (3)	C18—C19—H19	119.5
C8—C7A—C4A	108.7 (3)	C19—C20—C21	119.2 (3)
C7—C7A—C4A	101.0 (3)	C19—C20—H20	120.4
C8—C7A—H7C	108.4	C21—C20—H20	120.4
C7—C7A—H7C	108.4	C20—C21—C22	121.0 (4)
C4A—C7A—H7C	108.4	C20—C21—H21	119.5
C8A—C8—C7A	114.0 (3)	C22—C21—H21	119.5
C8A—C8—H8	123.0	C17—C22—C21	119.5 (4)
C7A—C8—H8	123.0	C17—C22—H22	120.3
C8—C8A—C3A	120.7 (3)	C21—C22—H22	120.3
C8—C8A—S1	128.0 (3)		
			
C8A—S1—C2—C3	0.8 (4)	C3—C3A—C8A—C8	−179.1 (4)
C8A—S1—C2—C9	177.4 (4)	C4—C3A—C8A—C8	−46.1 (5)
C9—C2—C3—C3A	−173.4 (4)	C3—C3A—C8A—S1	5.9 (4)
S1—C2—C3—C3A	2.9 (5)	C4—C3A—C8A—S1	139.0 (3)
C2—C3—C3A—C8A	−5.7 (5)	C2—S1—C8A—C8	−178.5 (4)
C2—C3—C3A—C4	−136.9 (4)	C2—S1—C8A—C3A	−4.0 (3)
C3—C3A—C4—C10	−88.3 (4)	C11—C4—C10—O1	143.8 (4)
C8A—C3A—C4—C10	144.2 (3)	C4A—C4—C10—O1	22.0 (5)
C3—C3A—C4—C11	36.3 (5)	C3A—C4—C10—O1	−91.4 (4)
C8A—C3A—C4—C11	−91.3 (4)	C11—C4—C10—O2	−42.6 (4)
C3—C3A—C4—C4A	156.0 (3)	C4A—C4—C10—O2	−164.4 (3)
C8A—C3A—C4—C4A	28.5 (4)	C3A—C4—C10—O2	82.2 (3)
C10—C4—C4A—C5	36.4 (4)	C10—C4—C11—C12	−32.8 (4)
C11—C4—C4A—C5	−89.2 (4)	C4A—C4—C11—C12	90.2 (4)
C3A—C4—C4A—C5	146.7 (3)	C3A—C4—C11—C12	−150.6 (3)
C10—C4—C4A—C7A	−88.3 (3)	C10—C4—C11—C16	156.6 (3)
C11—C4—C4A—C7A	146.1 (3)	C4A—C4—C11—C16	−80.3 (4)
C3A—C4—C4A—C7A	22.0 (4)	C3A—C4—C11—C16	38.9 (4)
C7A—C4A—C5—O3	161.3 (4)	C16—C11—C12—C13	0.2 (5)
C4—C4A—C5—O3	31.0 (6)	C4—C11—C12—C13	−170.6 (3)
C7A—C4A—C5—N6	−22.5 (4)	C11—C12—C13—C14	−0.7 (5)
C4—C4A—C5—N6	−152.8 (3)	C12—C13—C14—C15	0.9 (5)
O3—C5—N6—C17	0.1 (6)	C13—C14—C15—C16	−0.6 (5)
C4A—C5—N6—C17	−176.1 (3)	C14—C15—C16—C11	0.1 (5)
O3—C5—N6—C7	174.8 (4)	C12—C11—C16—C15	0.1 (5)
C4A—C5—N6—C7	−1.4 (4)	C4—C11—C16—C15	171.0 (3)
C5—N6—C7—C7A	24.6 (4)	C5—N6—C17—C22	−32.3 (6)
C17—N6—C7—C7A	−160.5 (3)	C7—N6—C17—C22	153.5 (4)
N6—C7—C7A—C8	−156.3 (3)	C5—N6—C17—C18	151.7 (4)
N6—C7—C7A—C4A	−36.3 (3)	C7—N6—C17—C18	−22.4 (5)
C5—C4A—C7A—C8	164.7 (3)	C22—C17—C18—C19	−0.8 (6)
C4—C4A—C7A—C8	−62.4 (4)	N6—C17—C18—C19	175.2 (4)
C5—C4A—C7A—C7	36.2 (3)	C17—C18—C19—C20	−0.4 (6)
C4—C4A—C7A—C7	169.1 (3)	C18—C19—C20—C21	1.4 (6)
C7—C7A—C8—C8A	163.6 (4)	C19—C20—C21—C22	−1.2 (6)
C4A—C7A—C8—C8A	47.5 (5)	C18—C17—C22—C21	1.1 (6)
C7A—C8—C8A—C3A	4.4 (5)	N6—C17—C22—C21	−174.8 (4)
C7A—C8—C8A—S1	178.4 (3)	C20—C21—C22—C17	−0.1 (6)

### (3aSR,4RS,4aRS,7aSR)-5-Oxothieno[2,3-f]isoindole-4-carboxylic acid (I) Hydrogen-bond geometry (Å, º)

**Table:**

*D*—H···*A*	*D*—H	H···*A*	*D*···*A*	*D*—H···*A*
O2—H2···O3^i^	1.04 (5)	1.63 (5)	2.667 (4)	174 (4)

Symmetry code: (i) −*x*+1/2, *y*+1/2, −*z*+1/2.

### (3aSR,4SR,4aRS,7aSR)-5-Oxofuro[2,3-f]isoindole-4-carboxylic acid (II) Crystal data

**Table:**

C_24_H_21_NO_4_	*Z* = 2
*M**_r_* = 387.42	*F*(000) = 408
Triclinic, *P*1	*D*_x_ = 1.294 Mg m^−^^3^
*a* = 8.1851 (16) Å	Synchrotron radiation, λ = 0.81182 Å
*b* = 11.025 (2) Å	Cell parameters from 600 reflections
*c* = 11.795 (2) Å	θ = 3.5–30.0°
α = 99.14 (3)°	µ = 0.12 mm^−^^1^
β = 92.51 (3)°	*T* = 100 K
γ = 107.99 (3)°	Prism, colourless
*V* = 994.6 (4) Å^3^	0.20 × 0.12 × 0.08 mm

### (3aSR,4SR,4aRS,7aSR)-5-Oxofuro[2,3-f]isoindole-4-carboxylic acid (II) Data collection

**Table:**

Rayonix SX165 CCD diffractometer	3839 reflections with *I* > 2σ(*I*)
/f scan	*R*_int_ = 0.092
Absorption correction: multi-scan (SCALA; Evans, 2006)	θ_max_ = 31.0°, θ_min_ = 3.4°
*T*_min_ = 0.963, *T*_max_ = 0.987	*h* = −10→10
18107 measured reflections	*k* = −13→13
4204 independent reflections	*l* = −14→14

### (3aSR,4SR,4aRS,7aSR)-5-Oxofuro[2,3-f]isoindole-4-carboxylic acid (II) Refinement

**Table:**

Refinement on *F*^2^	Secondary atom site location: difference Fourier map
Least-squares matrix: full	Hydrogen site location: mixed
*R*[*F*^2^ > 2σ(*F*^2^)] = 0.048	H atoms treated by a mixture of independent and constrained refinement
*wR*(*F*^2^) = 0.129	*w* = 1/[σ^2^(*F*_o_^2^) + (0.0543*P*)^2^ + 0.2912*P*] where *P* = (*F*_o_^2^ + 2*F*_c_^2^)/3
*S* = 1.06	(Δ/σ)_max_ < 0.001
4204 reflections	Δρ_max_ = 0.31 e Å^−^^3^
276 parameters	Δρ_min_ = −0.25 e Å^−^^3^
4 restraints	Extinction correction: SHELXL2014 (Sheldrick, 2015b), Fc^*^=kFc[1+0.001xFc^2^λ^3^/sin(2θ)]^-1/4^
Primary atom site location: difference Fourier map	Extinction coefficient: 0.060 (10)

### (3aSR,4SR,4aRS,7aSR)-5-Oxofuro[2,3-f]isoindole-4-carboxylic acid (II) Special details

**Table:**

Geometry. All esds (except the esd in the dihedral angle between two l.s. planes) are estimated using the full covariance matrix. The cell esds are taken into account individually in the estimation of esds in distances, angles and torsion angles; correlations between esds in cell parameters are only used when they are defined by crystal symmetry. An approximate (isotropic) treatment of cell esds is used for estimating esds involving l.s. planes.

### (3aSR,4SR,4aRS,7aSR)-5-Oxofuro[2,3-f]isoindole-4-carboxylic acid (II) Fractional atomic coordinates and isotropic or equivalent isotropic displacement parameters (Å^2^)

**Table:**

	*x*	*y*	*z*	*U*_iso_*/*U*_eq_	Occ. (<1)
O1	0.35211 (12)	−0.05879 (8)	0.66567 (7)	0.0201 (2)	
O2	0.5385 (4)	0.4663 (2)	0.8623 (2)	0.0241 (6)	0.6
O3	0.5530 (4)	0.35005 (19)	1.00161 (16)	0.0201 (5)	0.6
H3B	0.520 (4)	0.413 (3)	1.044 (3)	0.030*	0.6
O2'	0.5693 (7)	0.3756 (4)	0.9980 (2)	0.0241 (6)	0.4
O3'	0.5162 (7)	0.4567 (4)	0.8431 (3)	0.0201 (5)	0.4
H3C	0.480 (6)	0.505 (4)	0.901 (4)	0.030*	0.4
O4	0.87874 (12)	0.52722 (8)	0.76246 (7)	0.0223 (2)	
C2	0.35057 (15)	−0.07054 (12)	0.78383 (10)	0.0190 (3)	
C3	0.39805 (15)	0.04459 (11)	0.85592 (10)	0.0187 (3)	
H3	0.4076	0.0566	0.9378	0.022*	
C3A	0.43406 (15)	0.15241 (11)	0.78540 (10)	0.0169 (3)	
H3A	0.3397	0.1921	0.7944	0.020*	
C4	0.61590 (15)	0.26934 (10)	0.81143 (9)	0.0148 (2)	
C4A	0.66305 (14)	0.30967 (10)	0.69318 (9)	0.0145 (2)	
H4A	0.7364	0.2566	0.6627	0.017*	
C5	0.76917 (15)	0.44984 (11)	0.68806 (10)	0.0163 (3)	
N6	0.72662 (13)	0.46920 (9)	0.57808 (8)	0.0168 (2)	
C7	0.60501 (15)	0.34914 (11)	0.50551 (10)	0.0174 (3)	
H7A	0.6678	0.2984	0.4593	0.021*	
H7B	0.5233	0.3697	0.4533	0.021*	
C7A	0.51119 (14)	0.27614 (11)	0.59767 (10)	0.0155 (3)	
H7C	0.4223	0.3152	0.6266	0.019*	
C8	0.43213 (15)	0.12915 (11)	0.56824 (10)	0.0175 (3)	
H8	0.4044	0.0814	0.4916	0.021*	
C8A	0.40597 (15)	0.07445 (11)	0.66291 (10)	0.0165 (3)	
C9	0.30386 (19)	−0.20751 (12)	0.80397 (11)	0.0254 (3)	
H9A	0.1880	−0.2570	0.7663	0.038*	
H9B	0.3056	−0.2078	0.8871	0.038*	
H9C	0.3874	−0.2473	0.7717	0.038*	
C10	0.56971 (15)	0.37472 (10)	0.89451 (9)	0.0163 (3)	
C11	0.76528 (15)	0.23063 (11)	0.86340 (10)	0.0157 (3)	
C12	0.78551 (16)	0.10987 (11)	0.81712 (10)	0.0181 (3)	
H12	0.7030	0.0516	0.7581	0.022*	
C13	0.92622 (17)	0.07539 (12)	0.85763 (11)	0.0223 (3)	
H13	0.9373	−0.0063	0.8266	0.027*	
C14	1.04993 (17)	0.16108 (13)	0.94346 (11)	0.0243 (3)	
H14	1.1443	0.1373	0.9712	0.029*	
C15	1.03410 (17)	0.28250 (13)	0.98856 (11)	0.0235 (3)	
H15	1.1188	0.3415	1.0459	0.028*	
C16	0.89229 (16)	0.31656 (11)	0.94849 (10)	0.0189 (3)	
H16	0.8823	0.3987	0.9794	0.023*	
C17	0.81267 (15)	0.58110 (11)	0.53022 (10)	0.0175 (3)	
C18	0.87154 (17)	0.70563 (12)	0.60084 (11)	0.0216 (3)	
H18	0.8574	0.7155	0.6809	0.026*	
C19	0.95104 (17)	0.81456 (12)	0.55163 (12)	0.0250 (3)	
H19	0.9921	0.8980	0.5992	0.030*	
C20	0.97049 (17)	0.80153 (12)	0.43305 (12)	0.0249 (3)	
H20	1.0234	0.8759	0.4005	0.030*	
C21	0.91129 (16)	0.67809 (12)	0.36291 (11)	0.0226 (3)	
H21	0.9237	0.6690	0.2826	0.027*	
C22	0.83345 (15)	0.56730 (12)	0.41117 (11)	0.0197 (3)	
H22	0.7952	0.4838	0.3637	0.024*	

### (3aSR,4SR,4aRS,7aSR)-5-Oxofuro[2,3-f]isoindole-4-carboxylic acid (II) Atomic displacement parameters (Å^2^)

**Table:**

	*U*^11^	*U*^22^	*U*^33^	*U*^12^	*U*^13^	*U*^23^
O1	0.0251 (5)	0.0155 (4)	0.0175 (4)	0.0025 (3)	0.0019 (3)	0.0051 (3)
O2	0.0368 (12)	0.0244 (9)	0.0186 (11)	0.0183 (8)	0.0076 (8)	0.0064 (7)
O3	0.0347 (11)	0.0239 (10)	0.0123 (8)	0.0210 (9)	0.0104 (6)	0.0078 (6)
O2'	0.0368 (12)	0.0244 (9)	0.0186 (11)	0.0183 (8)	0.0076 (8)	0.0064 (7)
O3'	0.0347 (11)	0.0239 (10)	0.0123 (8)	0.0210 (9)	0.0104 (6)	0.0078 (6)
O4	0.0264 (5)	0.0184 (4)	0.0182 (4)	0.0030 (3)	0.0001 (4)	0.0016 (3)
C2	0.0186 (6)	0.0206 (6)	0.0189 (6)	0.0054 (4)	0.0048 (4)	0.0077 (4)
C3	0.0189 (6)	0.0211 (6)	0.0173 (6)	0.0065 (5)	0.0054 (4)	0.0059 (4)
C3A	0.0168 (5)	0.0173 (5)	0.0175 (6)	0.0057 (4)	0.0044 (4)	0.0042 (4)
C4	0.0173 (5)	0.0140 (5)	0.0140 (5)	0.0060 (4)	0.0042 (4)	0.0026 (4)
C4A	0.0161 (5)	0.0152 (5)	0.0140 (5)	0.0072 (4)	0.0036 (4)	0.0031 (4)
C5	0.0181 (6)	0.0163 (5)	0.0158 (6)	0.0072 (4)	0.0041 (4)	0.0026 (4)
N6	0.0194 (5)	0.0144 (5)	0.0169 (5)	0.0050 (4)	0.0029 (4)	0.0043 (4)
C7	0.0195 (6)	0.0168 (5)	0.0157 (5)	0.0049 (4)	0.0014 (4)	0.0043 (4)
C7A	0.0154 (5)	0.0165 (5)	0.0162 (5)	0.0064 (4)	0.0020 (4)	0.0048 (4)
C8	0.0172 (5)	0.0177 (5)	0.0168 (6)	0.0051 (4)	−0.0005 (4)	0.0024 (4)
C8A	0.0150 (5)	0.0143 (5)	0.0198 (6)	0.0040 (4)	0.0018 (4)	0.0033 (4)
C9	0.0323 (7)	0.0201 (6)	0.0227 (6)	0.0044 (5)	0.0040 (5)	0.0080 (5)
C10	0.0180 (6)	0.0171 (5)	0.0153 (6)	0.0075 (4)	0.0037 (4)	0.0034 (4)
C11	0.0184 (6)	0.0168 (5)	0.0144 (5)	0.0070 (4)	0.0061 (4)	0.0060 (4)
C12	0.0206 (6)	0.0181 (5)	0.0175 (6)	0.0084 (4)	0.0043 (4)	0.0034 (4)
C13	0.0268 (7)	0.0237 (6)	0.0232 (6)	0.0150 (5)	0.0070 (5)	0.0086 (5)
C14	0.0229 (6)	0.0361 (7)	0.0215 (6)	0.0165 (5)	0.0054 (5)	0.0121 (5)
C15	0.0198 (6)	0.0325 (7)	0.0177 (6)	0.0084 (5)	0.0011 (5)	0.0035 (5)
C16	0.0198 (6)	0.0200 (6)	0.0171 (6)	0.0069 (5)	0.0038 (4)	0.0020 (4)
C17	0.0155 (5)	0.0177 (5)	0.0222 (6)	0.0071 (4)	0.0037 (4)	0.0077 (4)
C18	0.0237 (6)	0.0190 (6)	0.0231 (6)	0.0074 (5)	0.0027 (5)	0.0054 (5)
C19	0.0237 (6)	0.0170 (6)	0.0346 (7)	0.0060 (5)	0.0028 (5)	0.0068 (5)
C20	0.0201 (6)	0.0230 (6)	0.0371 (7)	0.0089 (5)	0.0089 (5)	0.0154 (5)
C21	0.0203 (6)	0.0277 (6)	0.0260 (7)	0.0117 (5)	0.0088 (5)	0.0131 (5)
C22	0.0189 (6)	0.0210 (6)	0.0224 (6)	0.0091 (5)	0.0048 (5)	0.0070 (4)

### (3aSR,4SR,4aRS,7aSR)-5-Oxofuro[2,3-f]isoindole-4-carboxylic acid (II) Geometric parameters (Å, º)

**Table:**

O1—C8A	1.4034 (14)	C7A—H7C	1.0000
O1—C2	1.4203 (14)	C8—C8A	1.3460 (17)
O2—C10	1.226 (2)	C8—H8	0.9500
O3—C10	1.3378 (19)	C9—H9A	0.9800
O3—H3B	0.91 (3)	C9—H9B	0.9800
O2'—C10	1.219 (3)	C9—H9C	0.9800
O3'—C10	1.331 (3)	C11—C16	1.4083 (18)
O3'—H3C	0.91 (5)	C11—C12	1.4181 (16)
O4—C5	1.2339 (16)	C12—C13	1.4069 (17)
C2—C3	1.3447 (18)	C12—H12	0.9500
C2—C9	1.4994 (16)	C13—C14	1.399 (2)
C3—C3A	1.5202 (16)	C13—H13	0.9500
C3—H3	0.9500	C14—C15	1.4066 (19)
C3A—C8A	1.5274 (17)	C14—H14	0.9500
C3A—C4	1.6187 (17)	C15—C16	1.4104 (18)
C3A—H3A	1.0000	C15—H15	0.9500
C4—C11	1.5469 (16)	C16—H16	0.9500
C4—C10	1.5475 (15)	C17—C22	1.4118 (17)
C4—C4A	1.5590 (15)	C17—C18	1.4154 (18)
C4A—C5	1.5333 (16)	C18—C19	1.4052 (17)
C4A—C7A	1.5530 (17)	C18—H18	0.9500
C4A—H4A	1.0000	C19—C20	1.404 (2)
C5—N6	1.3943 (15)	C19—H19	0.9500
N6—C17	1.4339 (15)	C20—C21	1.404 (2)
N6—C7	1.4922 (16)	C20—H20	0.9500
C7—C7A	1.5455 (16)	C21—C22	1.4120 (17)
C7—H7A	0.9900	C21—H21	0.9500
C7—H7B	0.9900	C22—H22	0.9500
C7A—C8	1.5229 (16)		
			
C8A—O1—C2	106.64 (9)	O1—C8A—C3A	110.05 (10)
C10—O3—H3B	108.7 (19)	C2—C9—H9A	109.5
C10—O3'—H3C	105 (3)	C2—C9—H9B	109.5
C3—C2—O1	113.10 (10)	H9A—C9—H9B	109.5
C3—C2—C9	132.64 (11)	C2—C9—H9C	109.5
O1—C2—C9	114.21 (11)	H9A—C9—H9C	109.5
C2—C3—C3A	109.08 (10)	H9B—C9—H9C	109.5
C2—C3—H3	125.5	O2'—C10—O3'	122.8 (3)
C3A—C3—H3	125.5	O2—C10—O3	123.54 (18)
C3—C3A—C8A	101.04 (9)	O2'—C10—C4	122.2 (2)
C3—C3A—C4	119.58 (10)	O2—C10—C4	122.69 (16)
C8A—C3A—C4	113.14 (10)	O3'—C10—C4	114.8 (2)
C3—C3A—H3A	107.5	O3—C10—C4	113.54 (12)
C8A—C3A—H3A	107.5	C16—C11—C12	118.27 (11)
C4—C3A—H3A	107.5	C16—C11—C4	122.03 (10)
C11—C4—C10	113.00 (9)	C12—C11—C4	119.44 (10)
C11—C4—C4A	108.45 (9)	C13—C12—C11	120.79 (12)
C10—C4—C4A	112.78 (9)	C13—C12—H12	119.6
C11—C4—C3A	113.68 (9)	C11—C12—H12	119.6
C10—C4—C3A	102.35 (9)	C14—C13—C12	120.22 (11)
C4A—C4—C3A	106.37 (9)	C14—C13—H13	119.9
C5—C4A—C7A	104.03 (9)	C12—C13—H13	119.9
C5—C4A—C4	120.24 (10)	C13—C14—C15	119.79 (12)
C7A—C4A—C4	116.57 (9)	C13—C14—H14	120.1
C5—C4A—H4A	104.8	C15—C14—H14	120.1
C7A—C4A—H4A	104.8	C14—C15—C16	119.94 (12)
C4—C4A—H4A	104.8	C14—C15—H15	120.0
O4—C5—N6	126.58 (11)	C16—C15—H15	120.0
O4—C5—C4A	127.39 (10)	C11—C16—C15	120.97 (11)
N6—C5—C4A	105.85 (10)	C11—C16—H16	119.5
C5—N6—C17	124.82 (10)	C15—C16—H16	119.5
C5—N6—C7	112.14 (9)	C22—C17—C18	119.90 (11)
C17—N6—C7	121.96 (9)	C22—C17—N6	119.68 (11)
N6—C7—C7A	101.90 (9)	C18—C17—N6	120.39 (11)
N6—C7—H7A	111.4	C19—C18—C17	119.53 (12)
C7A—C7—H7A	111.4	C19—C18—H18	120.2
N6—C7—H7B	111.4	C17—C18—H18	120.2
C7A—C7—H7B	111.4	C20—C19—C18	120.80 (12)
H7A—C7—H7B	109.3	C20—C19—H19	119.6
C8—C7A—C7	118.78 (10)	C18—C19—H19	119.6
C8—C7A—C4A	108.15 (9)	C21—C20—C19	119.66 (12)
C7—C7A—C4A	100.28 (9)	C21—C20—H20	120.2
C8—C7A—H7C	109.7	C19—C20—H20	120.2
C7—C7A—H7C	109.7	C20—C21—C22	120.33 (12)
C4A—C7A—H7C	109.7	C20—C21—H21	119.8
C8A—C8—C7A	112.47 (10)	C22—C21—H21	119.8
C8A—C8—H8	123.8	C17—C22—C21	119.77 (12)
C7A—C8—H8	123.8	C17—C22—H22	120.1
C8—C8A—O1	126.48 (11)	C21—C22—H22	120.1
C8—C8A—C3A	123.47 (10)		
			
C8A—O1—C2—C3	0.41 (14)	C4—C3A—C8A—C8	−47.35 (15)
C8A—O1—C2—C9	−177.19 (10)	C3—C3A—C8A—O1	2.97 (12)
O1—C2—C3—C3A	1.57 (14)	C4—C3A—C8A—O1	132.04 (10)
C9—C2—C3—C3A	178.61 (13)	C11—C4—C10—O2'	−38.9 (3)
C2—C3—C3A—C8A	−2.68 (13)	C4A—C4—C10—O2'	−162.3 (3)
C2—C3—C3A—C4	−127.51 (11)	C3A—C4—C10—O2'	83.8 (3)
C3—C3A—C4—C11	27.00 (14)	C11—C4—C10—O2	137.8 (2)
C8A—C3A—C4—C11	−91.82 (11)	C4A—C4—C10—O2	14.3 (2)
C3—C3A—C4—C10	−95.20 (11)	C3A—C4—C10—O2	−99.5 (2)
C8A—C3A—C4—C10	145.99 (9)	C11—C4—C10—O3'	146.8 (3)
C3—C3A—C4—C4A	146.28 (10)	C4A—C4—C10—O3'	23.3 (3)
C8A—C3A—C4—C4A	27.46 (12)	C3A—C4—C10—O3'	−90.5 (3)
C11—C4—C4A—C5	−87.06 (12)	C11—C4—C10—O3	−47.5 (2)
C10—C4—C4A—C5	38.87 (14)	C4A—C4—C10—O3	−170.99 (17)
C3A—C4—C4A—C5	150.30 (10)	C3A—C4—C10—O3	75.12 (19)
C11—C4—C4A—C7A	145.69 (9)	C10—C4—C11—C16	−26.72 (14)
C10—C4—C4A—C7A	−88.38 (12)	C4A—C4—C11—C16	99.09 (12)
C3A—C4—C4A—C7A	23.04 (12)	C3A—C4—C11—C16	−142.82 (10)
C7A—C4A—C5—O4	162.69 (11)	C10—C4—C11—C12	159.31 (10)
C4—C4A—C5—O4	29.90 (17)	C4A—C4—C11—C12	−74.88 (13)
C7A—C4A—C5—N6	−21.89 (11)	C3A—C4—C11—C12	43.20 (14)
C4—C4A—C5—N6	−154.68 (10)	C16—C11—C12—C13	1.81 (17)
O4—C5—N6—C17	4.24 (19)	C4—C11—C12—C13	176.01 (10)
C4A—C5—N6—C17	−171.23 (10)	C11—C12—C13—C14	−0.89 (18)
O4—C5—N6—C7	172.53 (11)	C12—C13—C14—C15	−0.55 (18)
C4A—C5—N6—C7	−2.94 (12)	C13—C14—C15—C16	1.02 (19)
C5—N6—C7—C7A	26.50 (12)	C12—C11—C16—C15	−1.34 (17)
C17—N6—C7—C7A	−164.83 (9)	C4—C11—C16—C15	−175.38 (10)
N6—C7—C7A—C8	−154.90 (10)	C14—C15—C16—C11	−0.06 (18)
N6—C7—C7A—C4A	−37.46 (10)	C5—N6—C17—C22	145.02 (12)
C5—C4A—C7A—C8	161.73 (9)	C7—N6—C17—C22	−22.18 (16)
C4—C4A—C7A—C8	−63.41 (12)	C5—N6—C17—C18	−37.12 (17)
C5—C4A—C7A—C7	36.67 (10)	C7—N6—C17—C18	155.69 (11)
C4—C4A—C7A—C7	171.53 (9)	C22—C17—C18—C19	−0.28 (18)
C7—C7A—C8—C8A	159.62 (11)	N6—C17—C18—C19	−178.14 (11)
C4A—C7A—C8—C8A	46.40 (13)	C17—C18—C19—C20	0.93 (19)
C7A—C8—C8A—O1	−172.99 (10)	C18—C19—C20—C21	−0.6 (2)
C7A—C8—C8A—C3A	6.30 (16)	C19—C20—C21—C22	−0.37 (19)
C2—O1—C8A—C8	177.14 (12)	C18—C17—C22—C21	−0.67 (17)
C2—O1—C8A—C3A	−2.22 (12)	N6—C17—C22—C21	177.20 (10)
C3—C3A—C8A—C8	−176.42 (11)	C20—C21—C22—C17	1.00 (18)

### (3aSR,4SR,4aRS,7aSR)-5-Oxofuro[2,3-f]isoindole-4-carboxylic acid (II) Hydrogen-bond geometry (Å, º)

**Table:**

*D*—H···*A*	*D*—H	H···*A*	*D*···*A*	*D*—H···*A*
O3—H3*B*···O2^i^	0.91 (3)	1.79 (3)	2.692 (3)	176 (3)
O3′—H3*C*···O2′^i^	0.91 (5)	1.79 (5)	2.690 (6)	169 (4)

Symmetry code: (i) −*x*+1, −*y*+1, −*z*+2.
